# The Efficacy of Targeted Exercise on Gross Motor and Neuromuscular Performance in Survivors of Childhood Leukemia: A Pilot Study

**DOI:** 10.3389/fped.2022.891650

**Published:** 2022-05-11

**Authors:** Victoria Marchese, Kelly Rock, Teresa York, Kathryn Ruble, Vicki L. Gray

**Affiliations:** ^1^Department of Physical Therapy and Rehabilitation Science, School of Medicine, University of Maryland, Baltimore, MD, United States; ^2^Department of Pediatrics, School of Medicine, University of Maryland, Baltimore, MD, United States; ^3^Pediatric Oncology, The Johns Hopkins Hospital, Johns Hopkins Medicine, Baltimore, MD, United States

**Keywords:** pediatrics, cancer, children, oncology, physical therapy, rehabilitation, survivorship, intervention

## Abstract

**Objectives:**

This quasi-experimental study examined the efficacy of targeted exercise training on gross motor performance and neuromuscular impairments in survivors of childhood acute lymphoblastic leukemia (ALL CCS).

**Materials and Methods:**

Ten ALL CCS (median age: 10 years; range: 6–14 years) performed a 6-week training program three times per week (five in-person sessions), including a warm-up, total body stretching, progressive jump rope training, and a cool down. Gross motor performance (test of gross motor proficiency) and lower extremity rate of muscle activation (electromyography), joint torques (motion capture and force plate), and jump height (motion capture) were measured during a countermovement jump at baseline and post-training.

**Results:**

Post-training, ALL CCS demonstrated improvements in body coordination, strength and agilty, bilateral coordination, running speed and agility, and strength gross motor performance (mean change: 1.6–8.1; *p* < 0.05), the rate of muscle activation of the tibialis anterior and vastus lateralis muscles (mean change: 0.58–0.75; *p* < 0.05), hip and ankle joint torques (mean change: 0.07; *p* < 0.05), and jump height (mean change: 0.05; *p* < 0.05).

**Conclusion:**

This study demonstrated that targeted exercise training can improve gross motor performance and neuromuscular impairments in ALL CCS post-medical treatment.

## Introduction

With advances in medical treatment, 5-year survival rates for children with acute lymphoblastic leukemia (ALL) exceed 90% (1). However, short- and long-term effects of chemotherapy agents such as vincristine and methotrexate can cause neuromuscular impairments that affect muscle performance (2, 3). The medical treatment of ALL can lead to neuromuscular impairments including decreased neuromuscular activation, delayed muscle contraction initiation timing, reduced amplitude of the muscle activity, and decreased muscle force-generating capacity (4, 5). These treatments can also result in activity limitiations in gross motor performance including decreased balance, impaired coordination, and reduced movement speed and agility (3, 6–9). Body structure and function impairments and activity limitiations in survivors of childhood ALL (ALL CCS) extend well beyond the completion of medical treatment (5, 10, 11).

ALL CCS experience gross motor performance delays during a critical period of motor development. While ALL CCS' peers are developing muscle strength, balance control, and gross motor skills, many ALL CCS decline in these skills during medical treatment (7, 8). As a result, ALL CCS have difficulty keeping up with their peers in achieving physical activity recommendations and maintaining an active lifestyle (12, 13). Ultimately, ALL CCS are at an increased risk for developing obesity, metabolic syndrome, and early frailty at an earlier than expected age (14, 15). Thus, training programs that can reverse neuromuscular impairments and impaired gross motor performance are important for ALL CCS during childhood and adolescence, a sensitive period of motor development.

Over the past 20 years, there have been a limited number of exercise intervention programs designed for children with cancer and, more specifically, for ALL CCS (16). Most published programs have focused on general strengthening and aerobic activities because the programs have been designed for children with various types of cancer (16). To date, programs for survivors of childhood cancers have largely focused on adventure-based training, prospective surveillance models, health education programs, group physical activity programs, and computer game-based exercise programs (8, 17–19). These engaging programs have demonstrated feasibility and improvements in gross motor performance and physical activity with high satisfaction levels (8, 17–19). The encouraging results from these previous studies have offered the support for future studies to begin developing targeted training programs for the unique needs of specific types of childhood cancer survivors. Research supports training programs that incorporate functional tasks that target the neuromuscular system, such as jumping rope, improve muscle strength and gross motor performance including balance and speed and agility in children without health conditions (20, 21).

The purpose of this study was to examine the efficacy of a targeted neuromuscular training program that utilized jumping rope. We hypothesized that after the training program, ALL CCS would demonstrate improved gross motor performance, improved neuromuscular performance during a countermovement jump, measured by the rate of muscle activation and joint torques of the lower extremity, and jump height.

## Materials and Methods

This was a pilot quasi-experimental intervention study design.

### Participants

Study participants were recruited through flyers and in-person communication from the University of Maryland Medical Center Pediatric Oncology Clinic and the Johns Hopkins Medical, The Sidney Kimmel Comprehensive Cancer Center, Long-Term Survivors Program for participation in this study at the Department of Physical Therapy and Rehabilitation Science in the School of Medicine at the University of Maryland, Baltimore. Participants were included if the participants: (1) were children 6–17 years old; (2) completed medical treatment for ALL within the past 5 years (1–60 months); and (3) had the ability to communicate in English. Participants were excluded if the participants: (1) had a diagnosis of a neurological disorder such as cerebral palsy or Down syndrome; or (2) were currently receiving physical therapy services. This study was approved by the University of Maryland, Baltimore, Institutional Review Board. All participants provided informed assent and caregivers provided informed consent.

The Bruininks-Oseretsky Test of Motor Proficiency, 2nd edition (BOT-2) subtests were used to assess bilateral coordination, balance, running speed and agility, and strength at baseline and post-training (22). There were two standard composite tests calculated from these subtests, the body coordination composite test, which included the bilateral coordination and balance subtests, and the strength and agility composite test, which included the strength and running speed and agility subtests. Items were administered and scored according to standardized procedures (22). The BOT-2 has established interrater reliability and test-retest reliability for each subtest and each age group (22). The established mean for each subtest scale score is 15 with a standard deviation (SD) of 5, and the mean for each composite standard score is 50 with a standard deviation of 10 (22). Below average (BA) performance and well-below average (WBA) performance on the BOT-2 is defined as 1 SD and 2 SD below the mean, respectively, for subtest scale scores and composite standard scores (22).

The participants performed five trials of a countermovement jump before training (baseline) and after completing training (post-training). At the start of each trial, the participants stood on two adjacent force platforms (AMTI, Inc., Newton, MA, USA) in an upright position. The participant's arms were positioned at the side, and both hands were slightly anterior to the hips. The outline of the participant's feet was traced on a paper taped to the force platforms to ensure a similar start position for the trials at baseline and post-training. After a demonstration trial, participants were instructed to “jump as high as you can.” The jump was performed with a downward movement into a squat position followed by a push-off from force platforms into a jump.

Electromyography (EMG) activity was recorded using dual electrodes with a 2-cm interelectrode distance using surface EMG with a TeleMyo^TM^ DTS 24-channel wireless EMG system (Noraxon, USA Inc. Scottsdale, AZ, USA). Sensors placed bilaterally over the muscle belly of the lateral gastrocnemius, tibialis anterior, and vastus lateralis, muscles were according to SENIAM recommendations (23). EMG signals were collected with MyoResearch XP software (Noraxon, USA Inc., Scottsdale, AZ, USA) at a sampling rate of 1,500 Hz and later exported to MATLAB for analysis. After subtracting the mean DC bias, the EMG signals were rectified and high pass filtered at 20 Hz. The onset of the muscle activity was identified when the EMG amplitude was greater than two standard deviations above the baseline activity, which was calculated 500 milliseconds preceding movement onset. The rate of muscle activation was calculated as the EMG amplitude, from the onset to peak, and then was divided by the time duration from onset to peak muscle activity. Values were normalized by dividing by the peak EMG amplitude of each muscle during the task. The values for the right and left limbs were averaged.

The lower extremity joint torques and jump height were measured from reflective markers placed on the bilateral acromion, lateral epicondyles, ulnar styloid processes, greater trochanters, lateral condyles, lateral malleoli, 2nd metatarsal heads, interaural axes, and on the top of the head. Kinematic data were recorded with a ten-camera motion capture system (VICON, Los Angeles, CA, USA) at 150 Hz. Ground reaction forces were measured using dual force plates at 600 Hz and resampled to 120 Hz. The joint torque for the hips, knees, and ankles was estimated by inverse dynamics as described by Zatsiorsky (24). Peak joint torque of the hips, knees, and ankles were identified as the maximum value during the jump acceleration phase from the lowest point of the body center of mass (COM) to toe-off from the force plates. Joint torques were normalized to the height and weight of the participant. Ground reaction forces and kinematic data were filtered using a 10 Hz low-pass 4^th^ order Butterworth zero-lag filter (MATLAB filtfilt) (MathWorks, Natick, MA, USA). Data were bandpass filtered between 16–500 Hz. The jump height, measured in the sagittal plane, was calculated from the body COM and estimated using the method described by Winter (25). Jump height was calculated as the difference between peak COM height during the jump and COM height at the start of the jump and then normalized by the height of the participant.

### Intervention

Over a 6-week interval, each participant performed 18 training sessions, including five one-on-one sessions with a physical therapist twice the first week and once the second, third, and fifth weeks (Table 1). The first intervention occurred during the same day as the baseline assessment and the final intervention session occurred within a week of the post-training assessment. The participants were also instructed to perform the training program at home a total of three times per week (including the in-person visits) for the duration of the study (Table 1). The study participants were provided with an exercise diary to track the home exercise program that was reviewed at each in-person session. Each in-person session included: (1) discussion of home exercise program completion and progress; (2) discussion of session goals and short-term goals; (3) warm-up; (4) total body stretching; (5) exercise program; and (6) cool-down. The physical therapist provided verbal cues and feedback for proper body position, but only provided hands-on contact to maintain safety, not to physically assist with performing the movement. During each in-person session, the jumping rope program total duration, set duration, rest time, revolution count, and the complexity of jumping task was modified based on the participant's individualized progress and logged.

**Table 1:** Timeline of the intervention sessions.

	**Week 1**	**Week 2**	**Week 3**	**Week 4**	**Week 5**	**Week 6**
In-person PT	√√	√	√	——-	√	——-
Home-program	√	√√	√√	√√√	√√	√√√

The warm-up included walking for 5 min, fast enough to achieve an 11 on the 6–20 rate of perceived exertion scale (RPE). Stretching was then performed for trunk flexors, trunk extensors, trunk lateral flexors, hip flexors, hip extensors, hip internal and external rotators, knee flexors and extensors, ankle plantarflexors, and ankle dorsiflexors. Each stretch was performed one time and held for 15 s. The participants then performed the jumping rope intervention using a beaded jump rope (~$10 USD; BuyJumpRopes.net. Wenatchee, WA, USA). The participants were instructed on the proper use and mechanics of jumping the rope. The participants started with a 5-s bout of jumping with a 10-s rest break for 10 sets. The program progression goal was to achieve 15 s of jumping with a 30-s rest break for 15–20 sets. The session goal was to increase the number of times the rope revolved during the 15-s jumping period. The jump roping techniques used included: pre-traditional jumping (jumping over a stationary rope), traditional jumping, scissor jumping, side stradding, straddle cross, heel-toe, criss-cross, toe-touch, 360, can can, and long-rope jumping.

### Statistical Analysis

Statistical analysis was performed using SPSS version 26.0 (IBM Inc., Chicago, IL, USA). Due to the sample size and non-normality of data, two-sided Wilcoxon signed-rank tests were used to compare the differences between the baseline and post-training for the BOT-2 subscale scaled scores and composite standard scores, rate of activation of the muscles (lateral gastrocnemius, tibialis anterior, vastus lateralis), the joint torques (hip, knee, and ankle), and the jump height. For all statistical analyses, the level of significance was set at an alpha value of 0.05. Effect size (*r*) was calculated using the formula: $r=\frac{Z}{\sqrt{N}}$ (26). Effect sizes were considered “small” if *r* ≥ 0.10, “medium” if *r* ≥ 0.30, and “large” if *r* ≥ 0.60 (27).

## Results

The 10 participants (5 males; 5 females) had a median age of 10 years (range: 6–14 years), and a median time from completion of treatment 3.79 years (range 1.75–4.42 years). Of the 10 participants successfully referred to and enrolled in this study, 100% completed every scheduled session, including the baseline and post-training assessments and the five training sessions. Table 2 summarizes the elements of the jumping rope intervention progression that were increased or decreased during each of the in-person training sessions. Due to the COVID-19 pandemic, one child (participant 7) completed two of the five in-person training sessions using a live synchronous telehealth video conferencing format vs. coming to the research training facility. In addition, part of this participant's post-training assessment was completed virtually, but the laboratory-based measures of the countermovement jump and BOT-2 testing items requiring the balance beam and distance running were not performed. Therefore, complete data sets from nine participants were analyzed for the rate of muscle activation, jump height, joint torques, BOT-2 balance and running speed and agility subtests scale scores, and BOT-2 body coordination and speed and agility composite standard scores.

**Table 2:** In-person jumping rope training progression.

**Participant**	**Session 1**	**Session 2**	**Session 3**	**Session 4**	**Session 5**
1	–	↑TD	↑SD, ↑C	↑SD	↑C
2	–	↑SD, ↑C	↑SD, ↑C	↑SD, ↑C	↑SD
3	–	↑SD, ↑C, ↑RC	↑SD, ↑C, ↑RC	↑SD, ↓RT	↑SD, ↑RC
4	–	↑SD, ↓C, ↑RC	↑SD, ↑C, ↑RC	↑SD, ↑C, ↑RC	↑RC
5	–	↑RC	↑RC	↓RC	↑RC
6	–	↑SD, ↑C, ↑RC	↑RC	↑SD, ↑C, ↑RC	↑RC
7	–	↑SD, ↑C, ↑RC	↑SD, ↑C, ↓RT, ↑RC	↑RC	↑SD, ↑C, ↑RC
8	–	↑TD, ↑SD	↑RC	↑C, ↑RC	↑SD, ↑C, ↑RC
9	–	↑TD, ↑SD, ↑RC	↑SD, ↑RC	↑SD, ↑C, ↑RC	↑RC
10	–	↓C, ↑RC	↑RC	↑SD, ↑RC	↑C

*TD, total duration; SD, set duration; C, complexity; RT, rest time; RC, revolution count; ↑, increase; ↓, decrease*.

At the baseline assessment, seven ALL CCS presented with below average or well-below average BOT-2 composite scores of the body coordination and five of ALL CCS presented with impaired strength and agility standard composite scores (Table 3). ALL CCS demonstrated below average or well-below average BOT-2 standard composite scores of body coordination (*n* = 3), balance (*n* = 6), running speed and agility (*n* = 3), and strength (*n* = 6) at the baseline assessment (Table 3). The participants' individual BOT-2 scaled and standard composite scores at baseline and post-training assessments are reported in Table 3. After training, ALL CSS improved body coordination (mean change = 8.09; *p* < 0.001; *r* = 0.60; “large”), strength and agility (mean change = 3.06; *p* = 0.039; *r* = 0.53; “medium”), bilateral coordination (mean change = 3.60; *p* = 0.001; *r* = 0.63, “large”), running speed and agility (mean change = 1.60; *p* = 0.015; *r* = 0.52; “medium”), and strength (mean change = 3.06; *p* = 0.037; *r* = 0.51; “medium”) (Table 3). Overall, ALL CCS improved BOT-2 balance scaled scores; however, statistical significance was not achieved (mean change = 3.06; *p* = 0.146; *r* = 0.35; “medium”).

**Table 3:** Bruininks-Oserestrky test of motor proficiency, 2nd edition scores.

	**Scaled score**								**Standard score**			
	**Bilateral coordination**		**Balance**		**Running speed and agility**		**Strength**		**Body coordination**		**Strength and agility**	
	**Baseline**	**Post-training**	**Baseline**	**Post-training**	**Baseline**	**Post-training**	**Baseline**	**Post-training**	**Baseline**	**Post-training**	**Baseline**	**Post-training**
1	**7**	**10**	**7**	11	**9**	10	**6**	**8**	**30**	**37**	**34**	**37**
2	13	18	13	15	11	13	12	14	45	52	43	46
3	11	16	11	11	13	21	**5**	12	**38**	45	**35**	52
4	13	19	19	13	15	16	15	18	50	50	48	55
5	**7**	13	**4**	**7**	**6**	**7**	**4**	**8**	**30**	**39**	**31**	36
6	11	20	12	13	14	19	13	12	41	53	47	50
7	16	20	**8**	n/a	16	n/a	14	17	**40**	n/a	52	n/a
8	13	20	**7**	**9**	11	13	**7**	**7**	**38**	47	**36**	**39**
9	15	19	**6**	12	11	**10**	**9**	**7**	**37**	51	41	**38**
10	**2**	**9**	**8**	13	**8**	**8**	**3**	**5**	**29**	**39**	**28**	**30**
Mean	10.80	16.40*	**9.50**	11.56	11.40	13.00*	**8.80**	10.80*	**37.80**	45.89*	**39.50**	42.56*
SD	4.07	4.03	4.13	2.27	3.01	4.57	4.19	4.26	6.45	5.84	7.53	8.00
**BA**	20%	20%	50%	20%	30%	20%	30%	50%	40%	30%	40%	30%
**WBA**	10%	0%	10%	0%	0%	0%	30%	0%	30%	0%	10%	10%

***Bold** indicates score below average (BA); **bold and underlined** indicates score well-below average (WBA). n/a indicates not applicable*.

* *Indicates P < 0.05 for Wilcoxon signed-rank test between baseline and post-training*.

**Figure 1:**
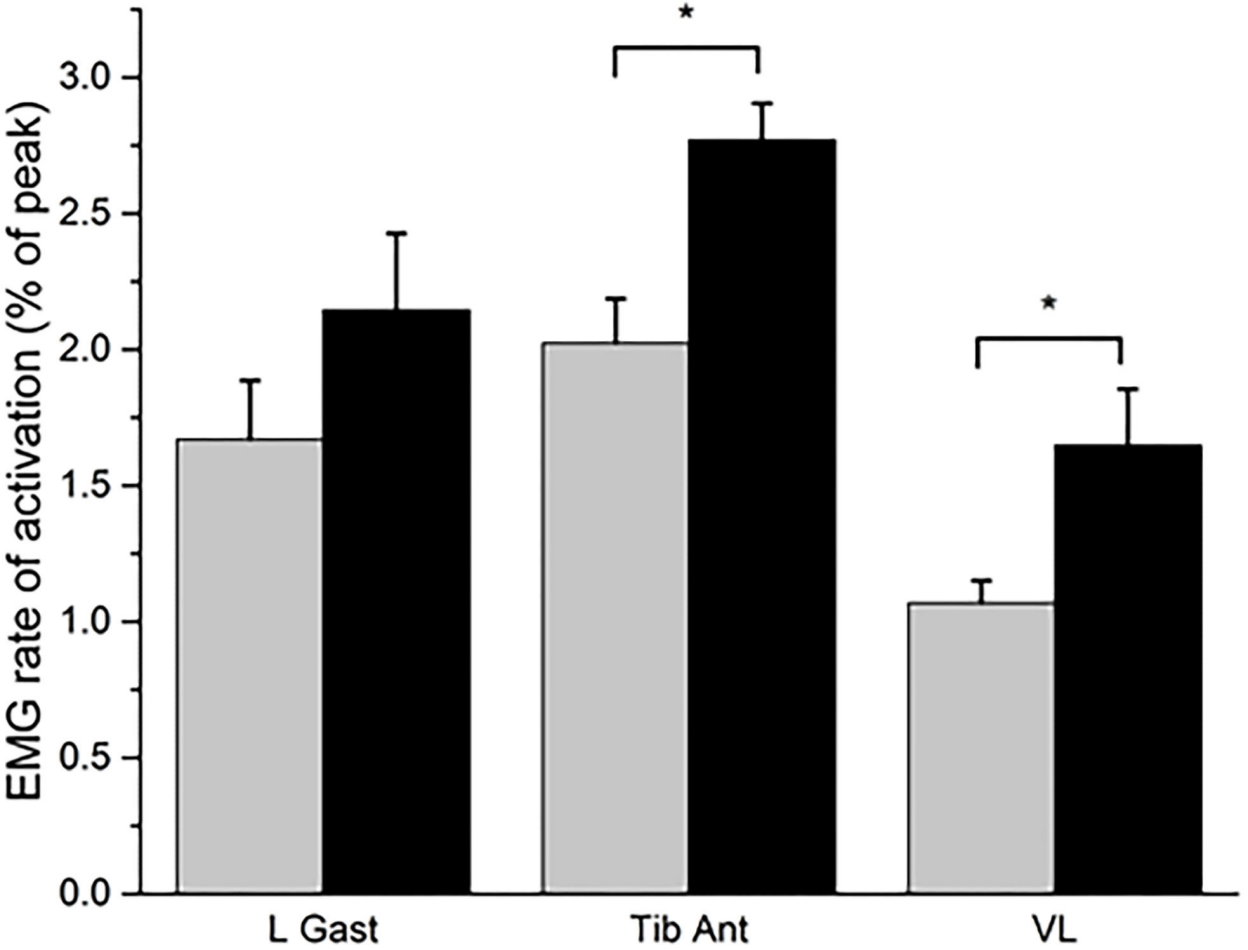
The rate of activation of the lateral gastrocnemius (L Gast), tibialis Anterior (Tib Ant), and vastus lateralis (VL) muscles at baseline (gray bar) and post-training (black bar). Expressed as the mean and standard error. *P < 0.05 for the Wilcoxon signed-rank test.

**Figure 2:**
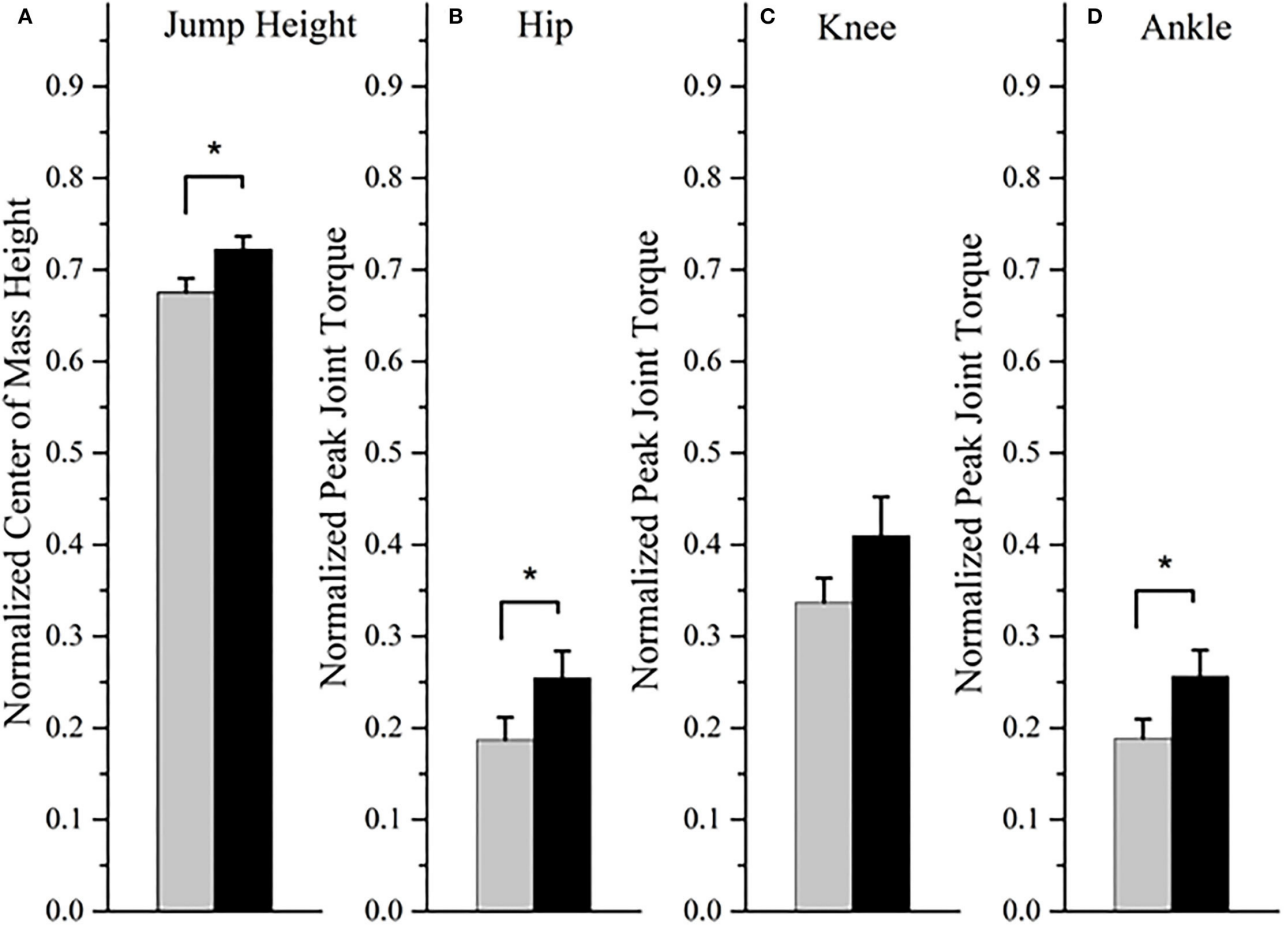
The jump height **(A)** and peak joint torque of the hip **(B)**, knee **(C)**, and ankle **(D)** at baseline (gray bar) and post-training (black bar). Expressed as the mean and standard error. *P < 0.05 for the Wilcoxon signed-rank test.

During the countermovement jump, there was a significant increase in the rate of activation of the tibialis anterior (mean change = 0.75; *p* = 0.008; *r* = 0.63; “large”) and vastus lateralis muscles (mean change = 0.58; *p* = 0.038; *r* = 0.49; “medium”) post-training compared to baseline (Figure 1). The rate of activation of the lateral gastrocnemius was not statistically significantly different after training (mean change = 0.47; *p* = 0.140; *r* = 0.35; “medium”). The joint torque of the hip (mean change = 0.07; *p* = 0.038; *r* = 0.49; “medium”) and ankle (mean change = 0.07; *p* = 0.008; *r* = 0.49; “medium”) significantly increased after training, along with the jump height (mean change = 0.05; *p* = 0.008; *r* = 0.89; “large”) (Figure 2). The knee joint torque did not significantly increase after training (mean change = 0.07; *p* = 0.069; *r* = 0.45; “medium”).

## Discussion

This targeted neuromuscular training program was feasible and improved gross motor and neuromuscular performance in ALL CCS post-training. In this study, these ALL CCS presented with baseline below average or well-below average BOT-2 standard composite scores of the body coordination (70%) and strength and agility (50%). Post-training the proportion of participants who performed below or well-below average decreased to 30% for body coordination and 40% for strength and agility, thus narrowing the gap in gross motor performance between these ALL CCS and a large normative sample of children without health conditions (22). Tanner & Hooke (8) reported similar findings with gross motor deficits in a cohort of ALL CCS and reported 47% and 73% of ALL CCS presented with body coordination and strength and agility composite scores within a healthy normative range, respectively (8). After this 6-week training, the participants in our study demonstrated significant improvements in gross motor performance, rate of tibialis anterior and vastus lateralis muscle activation, hip and ankle joint torque, and jump height. Previous randomized controlled trials have explored the effects of home exercise programs, including a standardized (28) and a progressive (29) flexibility, aerobic, and resistance training in ALL CCS post-treatment. These studies demonstrated improvements in gross motor performance and physical impairments including increased lower-extremity strength (28), decreased time to perform the Timed Up and Go (TUG) test (28, 29), decreased time to perform the Timed Up and Down Stairs (TUDS) test (28, 29), and increased distance on the 9-min run-walk test (28). However, Takken et al. (29, 30) found that after a community-based training program, no significant improvements were detected in hip, knee, ankle, or handgrip strength or performance on the TUG and TUDS. All of these interventions ranged from 12 weeks (28, 30) to 16 weeks (29). Our 6-week training, including five one-on-one sessions, shows that a shorter bout of targeted neuromuscular training using jumping rope can yield significant improvements in gross motor performance, neuromuscular activation, knee and ankle torque during a functional task, and jump height post-training in ALL CCS after completion of medical treatment.

We used specific targeted measurements to assess neuromuscular activation, lower extremity joint torque, and jump height during a countermovement jump in ALL CCS post-treatment including EMG and a motion capture system. These methods provide valuable information regarding the underlying neuromuscular mechanisms and whole-body movement that contribute to countermovement jump performance. Similarly, Wright, Twose, and Gorter also utilized EMG and motion capture to assess gait characteristics in ALL CCS (4) also utilized EMG and motion capture to assess gait characteristics in ALL CCS. In addition to impaired temporal-spatial, kinematic, and kinetic characteristics of gait in ALL CCS, the authors found gastrocnemius and tibialis anterior muscles excessive co-activation and atypical timing of EMG activation during the gait cycle (4). The use of EMG and motion capture in our study allowed for increased sensitivity to changes experienced by participants in response to the training. Although laboratory-based measurements of lower extremity joint torques during a jump and jump height in this study are challenging to use clinically, previous studies have demonstrated concurrent validity and reliability of 3D motion analysis with clinically-available measurements of height using an iPhone app in adults (31) and software paired with a traditional commercial camera in children (32). Therefore, measurements of jump height may be performed in clinical settings with minimal equipment.

Despite many benefits of this study, including the sensitivity of measurements and clinically-relevant outcome measures, this quasi-experimental intervention study lacked a control group; therefore, it is challenging to isolate the effects of spontaneous gross motor and neuromuscular improvements. Overall, ALL CCS improved gross motor and neuromuscular performance with medium to large effect sizes, however the small sample size may have led to type II error related to the efficacy of the intervention on balance, rate of activation of the lateral gastrocnemius, and knee joint torque. Due to the small sample size of ALL CCS from a single metropolitan area, caution should be practiced when generalizing these results. A randomized control trial should be explored to elucidate the effects of this training program further. Future studies should be performed to further explore if longer duration of training can provide additional benefit to improvements in gross motor and neuromuscular performance.

In conclusion, this feasible targeted neuromuscular training using jumping rope can improve gross motor and neuromuscular performance in ALL CCS post-medical treatment. The only equipment needed to perform this training was a beaded jump rope, which is a low-cost and long-lasting standard pediatric item in clinics, homes, and schools. Therefore, this training program has the potential to be easily implemented into current practices. The neuromuscular performance and motion capture methods used in this study are commonly used in research settings to identify movement system impairments. Still, this type of equipment is expensive and not readily available in most pediatric clinics, however, provides additional support of the efficiacy of this training program. However, in addition to gross motor performance testing, a vertical jump height during a counter movement jump can be measured and used as an indicator of rehabilitation progress in clinical settings.

## Data Availability Statement

The raw data supporting the conclusions of this article will be made available by the authors, without undue reservation.

## Ethics Statement

The studies involving human participants were reviewed and approved by University of Maryland, Baltimore Institutional Review Board. Written informed consent to participate in this study was provided by the participants' legal guardian/next of kin.

## Author Contributions

VM, TY, KRu, and VG contributed to the conception and design of the research. VM and VG performed the data acquisition and processing. VM, KRo, and VG analyzed and interpreted the data and drafted the manuscript. All authors contributed to the revision, critical appraisal, and approval of the manuscript submitted for publication.

## Funding

This study was supported in full by the American Physical Therapy Association Academy of Pediatric Physical Therapy Research Grant awarded to VM.

## Conflict of Interest

The authors declare that the research was conducted in the absence of any commercial or financial relationships that could be construed as a potential conflict of interest.

## Publisher's Note

All claims expressed in this article are solely those of the authors and do not necessarily represent those of their affiliated organizations, or those of the publisher, the editors and the reviewers. Any product that may be evaluated in this article, or claim that may be made by its manufacturer, is not guaranteed or endorsed by the publisher.
